# Radiation doses to mediastinal organs at risk in early-stage unfavorable Hodgkin lymphoma– a risk stratified analysis of the GHSG HD17 trial

**DOI:** 10.3389/fonc.2023.1183906

**Published:** 2023-05-05

**Authors:** Michael Oertel, Dominik Hering, Christian Baues, Christopher Kittel, Michael Fuchs, Jan Kriz, Kai Kröger, Dirk Vordermark, Klaus Herfarth, Rita Engenhart-Cabillic, Peter Lukas, Uwe Haverkamp, Peter Borchmann, Hans Theodor Eich

**Affiliations:** ^1^Department of Radiation Oncology, University Hospital Muenster, Muenster, Germany; ^2^Department of Radiation Oncology and Cyberknife Center, University Hospital of Cologne, Cologne, Germany; ^3^Department of Internal Medicine, Center for Integrated Oncology Aachen Bonn Cologne, Dusseldorf, University Hospital of Cologne, Cologne, Germany; ^4^Department of Radiation Oncology, Alexianer Clemenshospital Muenster, Muenster, Germany; ^5^Department of Radiation Oncology, University Hospital Halle (Saale), Halle (Saale), Germany; ^6^Department of Radiation Oncology, Heidelberg University Hospital, Heidelberg, Germany; ^7^Department of Radiotherapy and Radiation Oncology, University Hospital Giessen-Marburg, Marburg, Germany; ^8^Department of Radiooncology, Medical University Innsbruck, Innsbruck, Austria

**Keywords:** radiation oncology, radiation treatment, involved-node radiotherapy, involved-field radiotherapy, survivorship, dosimetry

## Abstract

**Introduction:**

The German Hodgkin Study Group (GHSG) HD17 trial established the omission of radiotherapy (RT) for patients with early-stage unfavorable Hodgkin lymphoma being PET-negative after 2 cycles of BEACOPP escalated plus 2 cycles of ABVD. This patient group reveals heterogeneity in characteristics and disease extent which prompted us to perform a decisive dosimetric analysis according to GHSG risk factors. This may help to tailor RT individually balancing risks and benefits.

**Methods:**

For quality assurance, RT-plans were requested from the treating facilities (n= 141) and analyzed centrally. Dose-volume histograms were scanned either paper-based or digitally to obtain doses to mediastinal organs. These were registered and compared according to GHSG risk factors.

**Results:**

Overall, RT plans of 176 patients were requested, 139 of which had dosimetric information on target volumes within the mediastinum. Most of these patients were stage II (92.8%), had no B-symptoms (79.1%) and were aged < 50 years (89.9%). Risk factors were present in 8.6% (extranodal involvement), 31.7% (bulky disease), 46.0% (elevated erythrocyte sedimentation rate) and 64.0% (three involved areas), respectively. The presence of bulky disease significantly affected the mean RT doses to the heart (p=0.005) and to the left lung (median: 11.3 Gy vs. 9.9 Gy; p=0.042) as well as V5 of the right and left lung, respectively (median right lung: 67.4% vs. 51.0%; p=0.011; median left lung: 65.9% vs. 54.2%; p=0.008). Significant differences in similar organs at risk parameters could be found between the sub-cohorts with the presence or absence of extranodal involvement, respectively. In contrast, an elevated erythrocyte sedimentation rate did not deteriorate dosimetry significantly. No association of any risk factor with radiation doses to the female breast was found.

**Conclusion:**

Pre-chemotherapy risk factors may help to predict potential RT exposure to normal organs and to critically review treatment indication. Individualized risk-benefit evaluations for patients with HL in early-stage unfavorable disease are mandatory.

## 1 Introduction

Patients with Hodgkin lymphoma (HL) reveal an excellent prognosis even in advanced stages (1–3). Consequently, attempts to reduce treatment intensity have been undertaken to minimize therapeutic burden aiming at an iso-effective de-escalation. This is of pivotal importance, as secondary malignancies and cardiovascular diseases attributed to therapy constitute the main mortality factors for long-term survivors of HL (4–8). Concerning radiation therapy (RT), modern guidelines describe a limitation to involved-site/involved-node (IN) RT volumes and treatment doses of 20-30 Gray (Gy) (9–11). The German Hodgkin Study Group (GHSG) has defined three distinct categories (early-stage favorable, early-stage unfavorable, advanced) according to Ann-Arbor stage and the presence of risk factors (mediastinal bulk, extranodal involvement, elevated erythrocyte sedimentation rate (ESR), ≥ 3 involved lymph node areas) guiding treatment and thereby also the use of RT (10). For early-stage favorable disease, randomized trials failed to establish a chemotherapy-only regimen without deterioration of prognosis (2, 12, 13). However, for early-stage unfavorable disease, the picture is more complex. The unfavorable arm of the European Organisation for Research and Treatment of Cancer (EORTC) H10 trial included HL-patients 15 to 70 years of age with at least one EORTC risk factor (age ≥ 50 years, ≥ 3 lymph node areas, elevated ESR, mediastinal bulk) (13). Patients were randomized after 2 chemotherapy cycles of doxorubicin, bleomycin, vinblastine, and dacarbazine (ABVD) between a standard approach including mandatory INRT and an experimental concept, in which no RT was administered to positron emission tomography (PET)-negative patients after chemotherapy. This chemotherapy-only arm was closed pre-maturely due to an increased number of events in an interim analysis (14). Final prognostic results for PET-negative patients were similar (5-year progression-free survival (PFS): 92.1% vs. 89.6%; 5-year overall survival: 96.7% versus 98.3% for the combined modality treatment-arm including RT and the chemotherapy-only arm, respectively), but non-inferiority of the monomodal treatment could not be demonstrated (13). Correspondingly, the GHSG HD17 trial introduced a PET-guided strategy for patients with early-stage unfavorable HL limiting RT to PET-positive patients (15). The 5-year PFS between this approach and the standard arm (using RT independent from PET-status) did not differ significantly (97.3% vs. 95.1%) and enabled the omission of RT for PET-negative patients after systemic therapy. Data on quality control and overall dosimetry have been published by our group (16) but heterogeneity of disease extent within early-stage unfavorable HL demands a decisive sub-analysis. Patients within this stage may show anatomic risk factors (bulk, extranodal involvement, ≥ 3 lymph node areas) but also mere blood level changes (elevated ESR), thus differences in disease extent and RT treatment volumes are to be expected. The current analysis reflects dosimetric data assessed by the radiation oncology reference panel of the GHSG investigating the impact of risk factors on dose exposure to different organs at risk in the mediastinum. A critical appraisal of these data is provided enabling a comparison to other study groups and pointing towards further developments.

## 2 Materials and methods

### 2.1 Study design and radiation treatment

The HD17 trial (NCT01356680; Eudract-code: 2007-005920-34) was conducted by the GHSG as a randomized phase III trial including patients in early-unfavorable stage and an age of 18-60 years (15). Patients received two cycles dose-escalated etoposide, cyclophosphamide and doxorubicin and regular doses of bleomycin, vincristine, procarbazine and prednisone and two cycles of ABVD. In the standard arm, all patients were treated with 30 Gy involved-field RT (IFRT) irrespective of the PET-status after completion of systemic therapy. In the experimental arm, a fluor-desoxy-glucose (FDG)-PET scan after chemotherapy guided further RT. PET-negative patients (Deauville-Score: 1-2) did not receive additional treatment. PET-positive patients (Deauville-Score: 3-5) were irradiated with INRT, the concept of which has been described previously (17). In summary, a clinical target volume was defined including the initial lymphoma extent but considering the displacement of healthy organs at risks. An expansion margin of 2 cm in axial and 3 cm in craniocaudal direction was applied to receive the planning target volume which was treated with 30 Gy in normofractionation. IFRT in the standard arm included irradiation of the involved mediastinum with safety margins according to the study protocol. For RT of the upper mediastinum (above the tracheal bifurcation), the field was delineated as follows: the hyoid bone constituted the cranial border and the supraclavicular region (medial 2/3 of the clavicula) down to the mediastinum was included, with the caudal border being one vertebra below the bifurcation without inclusion of the lung hili (unless involved). For RT of the lower mediastinum, the RT field began one vertebra below the bifurcation and ended at the level of the diaphragm (thoracic vertebra 10/11). Involved hili were treated with a safety margin of 1.5 cm, whereas the processus transversus constituted the lateral border if lung hili were not involved. All participants gave informed consent before participation and all procedures were executed in accordance with the Declaration of Helsinki.

### 2.2 Dosimetric analysis

Radiotherapy quality analysis was an integral part of the study protocol and covered by the institutional review board consent. The reference radiation oncology formulated treatment recommendations taking into account initial disease extent and response to systemic therapy as assessed by computed tomography (CT) and PET-CT scans before and after chemotherapy, respectively. After completion of RT, a random selection of 50 IFRT-plans and all INRT-plans were requested for quality analysis. These plans were requested from the treating facilities (n= 141) and analyzed centrally. This included a decisive evaluation of RT planning and execution by the reference radiation oncology panel of the GHSG. Additionally, dose-volume histograms (DVH) were analyzed regarding different organs at risks (OAR). For the current study, the dosimetric analysis has been limited to patients with irradiation treatments in the upper and/or lower mediastinum (above/below the tracheal bifurcation), respectively.

### 2.3 Statistical analysis

Numeric variables were summarized by the minimum, mean, median and maximum values, respectively. For comparison between different categories, a two-sample t-test or a Mann-Whitney-U-test were used depending on the presence of a normal distribution. Normal distributions were assessed *via* a Shapiro-Wilk test. Distributions between the subgroups were compared using a Kolmogorov-Smirnov test. Correlations between metric values were estimated using Pearson’s p. All statistical tests were considered significant with a p-value of ≤ 0.05. Analyses were carried out using Microsoft Excel (Microsoft, Redmond, WA, USA) and SPSS version 29 (IBM, Armonk, NY, USA).

## 3 Results

### 3.1 Patient collective

Overall, 139 patients with mediastinal RT within the HD17 collective could be identified, the majority of which (77.0%) belongs to the involved-node group. Mean doses to the planning target volume (PTV) were 5.3-34.2 Gy (median: 30.0 Gy). RT-techniques were predominantly 3D-conformal or APPA (54.0%), in comparison to advanced approaches like intensity modulated RT (IMRT), volumetric arc therapy or tomotherapy (46.0% taken together). Ann-Arbor stage was I in 7.2% and II in 92.8% of patients. Patients’ age was predominantly under 50 years (89.9% vs. 7.2% > 50 years; 2.9% unknown); none of the included patient was 60 years or older (97.1% < 60 years; 2.9% unknown). Concerning GHSG risk factors, an involvement of at least three lymph node areas, an elevated ESR, bulky disease and extranodal spread were present in 64.0%, 46.0%, 31.7%, 8.6%. Multiple risk factors were present in 45.3% of patients (2: 41.0%, 3: 3.6% and 4: 0.7%). B-symptoms were reported in 20.9% of patients.

### 3.2 Dosimetry

**Figure 1:**
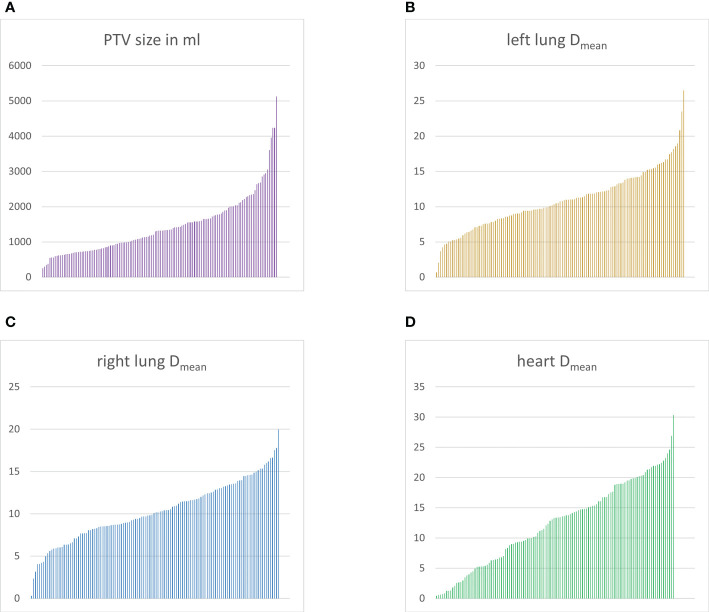
Overview of planning target volume (PTV) sizes and doses to mediastinal organs at risks. Bar graphs depicting every patient as a single bar sorted according to increasing values. PTV sizes **(A)** in ml, mean doses to the left lung **(B)**, right lung **(C)** and heart **(D)** in Gray, respectively.

PTV sizes differed between 252.6 ml and 5125.3 ml (**Figure 1A**). There was no significant difference in PTV size between patients treated with 3D-conformal or APPA techniques in comparison to advanced techniques (p=0.739). Mean doses to the heart, right and left lung, respectively were registered (**Figures 1B–D**) with resulting values of 0.5 Gy- 30.4 Gy (median: 13.3 Gy), 0.3-20.0 Gy (median: 10.1 Gy) and 0.7-26.5 Gy (median: 10.8 Gy), respectively. A detailed overview on OAR dosimetry is provided in **Table 1**. There was a significant correlation between PTV volume and mean lung dose (r=0.423 and r=0.442 for right and left lung, respectively; p< 0.001) as well as mean heart dose (r=0.417; p< 0.001). No significant correlation existed between PTV size and mean dose to the right (p=0.601) or left breast (p=0.654), respectively. Comparing the IFRT- and INRT-cohorts, most OAR parameters did not show significant differences except for the V25 in both lungs (right lung: p=0.042; left lung: median: 13.3 Gy with INRT vs. 17.8 Gy with IFRT; p= 0.018), maximum dose to the spinal cord spinal cord (p<0.001) and mean dose to the esophagus (p=0.044). Except for V25 of the left lung, all these comparisons showed significant differences in the Kolmogorov-Smirnov test between the INRT- and IFRT-group (V25 right lung: mean rank 54.01 with INRT vs. 68.76 with IFRT; U=870.5; Z=-2.027; spinal cord: mean rank 58.0 with INRT vs. 90.25 with IFRT; U=707.0; Z=-4.039; esophagus: mean rank 18.28 with INRT vs. 27.86 with IFRT; U=57.0; Z=-2.013). Consequently, both cohorts were pooled for subsequent analyses except for the parameters mentioned.

**Table 1:** Dosimetry of the overall study population.

OAR	parameter	n	Min.	Max.	Median
right lung	*Dmean (Gy)*	122	0.3	20	10.1
	*V5 (%)*	114	0	100	56
	*V20 (%)*	114	0	50	21
	*V25 (%)*	114	0	42	14
	*V30 (%)*	114	0	31.9	2
left lung	*Dmean (Gy)*	123	0.7	26.5	10.8
	*V5 (%)*	115	0.9	99	58
	*V20 (%)*	115	0	85	21
	*V25 (%)*	115	0	80	14.9
	*V30 (%)*	115	0	60	2
Myelon	Dmax (Gy)	129	6.9	34.2	29.7
Esophagus	Dmean (Gy)	39	10.1	30	21.6
Heart	Dmean (Gy)	118	0.5	30.4	13.3
right breast	Dmean (Gy)	27	0.5	9.3	3.7
left breast	Dmean (Gy)	26	0.4	15.6	3.6

Gy, Gray; Max., Maximal value; Min., Minimal value; OAR, organ at risk.

### 3.3 Risk stratified dosimetry according to GHSG risk factors

Relevant dosimetric parameters were analyzed according to the presence or absence of the GHSG risk factors: mediastinal bulk, extranodal involvement, three or more lymph node areas and elevated ESR (**Tables 2**–**5**). There was a difference in PTV sizes only between the subgroups with or without an involvement of three nodal areas (mean rank: 77.40 with vs. 46.79 without involvement; U=1071.0; Z=-4.403; p<0.001).

**Table 2:** Dosimetric impact of the presence or absence of bulky disease.

OAR	with bulk	Mean	Min.-Max.	without bulk		Min.-Max.	p
	Median			Median	Mean		
right lung							
*mean dose*	11.5	10.8	0.3-17.8	9.7	10.1	3.2-20.0	0.278
*V5 (%)*	67.4	64.6	0-98.3	51.0	53.7	12.4-100.0	**0.011**
*V20 (%)*	21.0	20.4	0-46.0	21.0	21.0	3.1-50.0	0.977
*V30 (%)*	2.0	4.2	0.0-30.0	2.0	4.4	0.0-31.9	0.348
left lung							
*mean dose*	11.3	12.0	2.0-26.5	9.9	10.4	0.7-20.8	**0.042**
*V5 (%)*	65.9	65.5	11.0-97.0	54.2	54.1	0.9-99.0	**0.008**
*V20 (%)*	23.8	24.2	0.0-85.0	21.0	21.7	0.2-48.0	0.656
*V30 (%)*	2.3	5.4	0.0-60.0	2.0	4.2	0.0-20.0	0.716
heart (mean)	15.1	14.9	1.3-30.4	10.0	11.2	0.5-26.9	0.005
breasts							
*left (mean)*	3.9	5.0	0.6-15.6	3.5	4.4	0.4-10.9	0.938
*right (mean)*	4.0	4.3	1.5-9.3	3.2	3.4	0.5-8.9	0.388

P-values are given for the Mann-Whitney U or two-sample t-test, respectively, according to the presence of a normal distribution.Significant values are indicated in bold numbers. Gy, Gray; OAR, organ at risk.

**Table 3:** Dosimetric impact of the presence or absence of extranodal disease.

OAR	with extranodal		Min.-Max.	without extranodal		Min.-Max.	p
	Median	Mean		Median	Mean		
right lung							
*mean dose*	12.6	13.5	6.4-20.0	9.8	10.0	0.3-17.5	**0.002**
*V5 (%)*	67.8	71.6	36.0-100.0	54.8	55.8	0.0-98.3	**0.028**
*V20 (%)*	25.3	26.0	4.1-50.0	20.5	20.3	0.0-45.0	0.088
*V30 (%)*	2.3	5.4	0.0-29.0	2.0	4.2	0.0-31.9	0.865
left lung							
*mean dose*	14.1	14.5	5.3-26.5	10.4	10.6	0.7-20.8	0.081
*V5 (%)*	91.0	79.1	30.0-99.0	56.0	55.9	0.9-97.0	**0.002**
*V20 (%)*	27.0	34.2	5.9-85.0	21.0	21.4	0.0-51.8	0.140
*V30 (%)*	1.5	10.0	0.0-60.0	2.0	4.1	0.0-21.0	0.506
heart (mean)	21.1	19.0	6.4-30.4	12.6	11.8	0.5-26.9	0.003

P-values are given for the Mann-Whitney U or two-sample t-test, respectively, according to the presence of a normal distribution. Significant values are indicated in bold numbers. Gy, Gray; OAR, organ at risk.

**Table 4:** Dosimetric impact of the presence or absence of three or more lymph node areas.

OAR	with 3 areas		Min.-Max.	without 3 areas		Min.-Max.	p
	Median	Mean		Median	Mean		
right lung							
*mean dose*	10.1	10.2	3.2-17.5	10.6	10.5	0.3-20.0	0.709
*V5 (%)*	55.0	55.8	12.4-98.0	59.0	59.9	0.0-100.0	0.336
*V20 (%)*	21.2	21.2	3.1-42.0	18.6	20.0	0.0-50.0	0.569
*V30 (%)*	2.0	3.7	0.0-20.0	2.0	5.4	0.0-31.9	0.951
left lung							
*mean dose*	11.0	11.3	4.3-26.5	10.1	10.1	0.7-23.5	0.108
*V5 (%)*	58.0	58.8	19.0-98.5	59.8	56.2	0.9-99.0	0.566
*V20 (%)*	23.3	24.5	5.0-85.0	18.6	18.9	0.0-70.3	**0.012**
*V30 (%)*	2.0	5.0	0.0-60.0	2.0	4.0	0.0-30.0	0.179
heart (mean)	11.8	12.0	0.6-26.9	13.6	13.2	0.5-30.4	0.435
breasts							
*left (mean)*	3.5	4.4	0.6-10.9	3.9	5.0	0.4-15.6	0.897
*right (mean)*	4.5	4.2	0.5-9.3	3.2	3.1	0.5-5.1	0.188

P-values are given for the Mann-Whitney U or two-sample t-test, respectively, according to the presence of a normal distribution. Significant values are indicated in bold numbers. Gy, Gray; OAR, organ at risk.

**Table 5:** Dosimetric impact of the presence or absence of an elevated erythrocyte sedimentation rate.

OAR	with ESR		Min.-Max.	without ESR		Min.-Max.	p
	Median	Mean		Median	Mean		
right lung							
*mean dose*	9.4	9.8	3.2-15.0	10.3	10.7	0.3-20.0	0.187
*V5 (%)*	49.5	54.7	12.4-94.5	57.5	59.4	0.0-100.0	0.259
*V20 (%)*	19.7	19.4	3.1-45.0	21.8	22.0	0.0-50.0	0.16
*V30 (%)*	1.5	3.9	0.0-31.9	2.3	4.6	0.0-30.0	0.331
left lung							
*mean dose*	9.9	9.9	0.7-18.2	11.5	11.8	2.0-26.5	**0.011**
*V5 (%)*	48.0	52.4	0.9-97.0	62.8	62.6	11.0-99.0	**0.012**
*V20 (%)*	20.0	19.8	0.2-48.0	23.4	24.8	0.0-85.0	0.087
*V30 (%)*	2.0	3.1	0.0-17.3	2.3	5.9	0.0-60.0	0.093
heart (mean)	12.9	11.4	0.5-21.7	14.1	13.4	0.6-30.4	0.119
breasts							
*left (mean)*	3.4	3.9	0.4-10.9	3.7	5.4	1.8-15.6	0.311
*right (mean)*	4.1	4.3	0.5-9.3	3.0	3.2	0.7-6.4	0.273

P-values are given for the Mann-Whitney U or two-sample t-test, respectively, according to the presence of a normal distribution. Significant values are indicated in bold numbers. ESR, erythrocyte sedimentation rate; Gy, Gray; OAR: organ at risk.

Bulky disease was associated with an increase in V5 of the right and left lung, respectively (median right lung: 67.4% vs. 51.0%; p=0.011; median left lung: 65.9% vs. 54.2%; p=0.008), mean dose to the left lung (median: 11.3 Gy vs. 9.9 Gy; p=0.042) and mean heart dose (p=0.005; **Table 2**). The latter revealed a significant different distribution in both groups in the Kolmogorov-Smirnov test (mean rank: 71.75 with bulky disease vs. 53.22 without; U=1070.0 Z=-2.786). Similar results were found regarding extranodal disease, with significant differences for mean dose and V5 of the right lung (12.6 Gy vs. 9.8 Gy; p=0.002 and 67.8% vs. 54.8%; p=0.028; **Table 3**). Additionally, there were significant differences in V5 to the left lung and mean heart dose, but with different distributions in each sub-group as assessed by the Kolmogorov-Smirnov test (V5 left lung: mean rank 87.85 with vs. 55.16 without extranodal involvement; U=226.5; Z=-2.963; p=0.002; mean heart dose: mean rank 89.10 with vs. 56.76 without extranodal involvement; U=244.0; Z=-2.860; p=0.003). The presence of three lymph node areas or more only affected the V20 for the left lung (**Table 4**), again with a different distribution between both groups (Kolmogorov-Smirnov: mean rank with three areas: 63.67 vs. 47.36 without three areas; U=1074.5; Z=-2.499; p=0.012). In contrast, an elevated ESR did not herald an increase in any OAR parameter examined (**Table 5**).

Analysis of multiple risk factors was restricted due to limited patient numbers (extranodal disease and bulk: 5 patients, extranodal disease and three lymph node areas: 4 patients). For bulky disease and three lymph node areas (15 patients), dosimetric comparisons were conducted (**Table 6**). A comparison with patients without this risk factor combination showed a significant difference in mean heart dose and V20 to the right and left lung, respectively but with different distributions as assessed by the Kolmogorov-Smirnov test (V20 left lung: mean rank 76.89 with vs. 55.38 without; U=442.5; Z=-2.263; p=0.023; V20 right lung: mean rank 76.89 with vs. 54.79 without; U=428.5; Z=-2.344; p=0.018; mean heart dose: mean rank 80.87 with vs. 56.39 without; U=452.0; Z=-2.589; p=0.009).

**Table 6:** Dosimetric impact of the presence or absence of bulky disease in combination with the involvement of three lymph node areas.

OAR	with bulk and 3 areas		Min.-Max.	without bulk and 3 areas		Min.-Max.	p
	Median	Mean		Median	Mean		
right lung							
*mean dose*	11.6	11.7	5.0-16,6	9.9	10.1	0.3-20.0	0.128
*V5 (%)*	58.8	62.4	29.8-94,0	55.0	56.5	0.0-100.0	0.342
*V20 (%)*	25.0	25.2	10.5-35,1	20.0	20.2	0.0-50.0	**0.018**
*V30 (%)*	2.8	4.5	0.0-11,0	2.0	4.3	0.0-31.9	0.351
left lung							
*mean dose*	11.9	12.9	4.3-26,5	10.5	10.6	0.7-23.5	0.056
*V5 (%)*	64.3	64.8	37.5-97,0	58.0	56.9	0.9-99.0	0.209
*V20 (%)*	27.5	30.8	5.0-85,0	20.1	21.4	0.0-70.3	**0.023**
*V30 (%)*	3.2	8.1	0.0-60,0	2.0	4.1	0.0-30.0	0.208
heart (mean)	19.0	16.5	1.8-22,8	11.5	11.8	0.5-30.4	**0.009**

P-values are given for the Mann-Whitney U or two-sample t-test, respectively, according to the presence of a normal distribution. Significant values are indicated in bold numbers. Gy, Gray; OAR, organ at risk.

## 4 Discussion

The presented analysis is one of the first to investigate an individualized risk-benefit evaluation for RT in early-stage Hodgkin lymphoma. Based on a large multicenter trial, it provides a unique quality-controlled analysis of known risk factors and identifies significant predictors for higher dose exposure to normal tissue. Whereas an elevated ESR did not affect OAR dosimetry, bulky disease or extranodal involvement were associated with higher cardiopulmonary dose exposure. The planning target volume was extended significantly by the presence of at least three involved lymph node areas. The aforementioned categories challenge established dose constraints and demand for a careful RT planning balancing putative (long-term) toxicity with efficacy.

With cardiovascular disease and secondary neoplasia being the predominant causes of death for long-term survivors of HL (4–8), RT dose exposure to mediastinal organs at risk (OAR) is of cardinal importance. Concerning the heart, RT may lead to a spectrum of diseases like cardiomyopathy, coronary heart disease (CHD), valvular dysfunction, conduction disorders, pericardial effusion or inflammation (18). Importantly, cardiac substructures reveal a different response to radiation exposure with CHD being linearly correlated to the mean heart dose; whereas valvular diseases augment exponentially beyond 30 Gy (19, 20). In the lungs, RT induces a complex damage pattern to the alveolar epithelium involving cell-senescence, DNA-damage, (sub-)acute inflammation (pneumonitis) and chronic consecutive lung fibrosis (18).

Individual doses to mediastinal OAR may vary considerably as demonstrated by analyses of the UK RAPID (21) and the HD17 (16) trial. With a radiation dose of 30 Gy, the resulting heart exposure differed between 0.1-24 Gy (21) and 0.5-30.4 Gy (16), which leads to heterogenous long-term risks. This is also true for the mediastinal sub-cohort discussed here (**Figure 1D**). Overall, the International Lymphoma Radiation Oncology Group (ILROG) recommends to reduce mean heart, breast and lung doses below 5, 4 and 10 Gy respectively (22). However, these constraints are only respected by a minority of cases in our analysis. Interestingly, bulky disease and extranodal spread significantly impacted low-dose exposure to the lung, which may be associated with an rise in secondary lung cancers as shown by biophysical risk evaluations (23, 24). Consequently, the ILROG suggests to keep respective doses below 55%, with a V5 > 60% being unacceptable (22). Both the presence of extranodal disease or bulky disease led to a low-dose exposure exceeding this threshold, demanding for a careful plan evaluation by the treating radiation oncology facility. In contrast, no significant correlation between GHSG risk factors and mean breast dose could be outlined in our analysis, with the ILROG constraints being met in most subcategories (**Tables 2**–**5**). Nonetheless, even low-dose exposure of the breast may lead to an excessive risk of carcinogenesis, particular in young patients with vulnerable glandular tissue (24, 25). As a result, a rational strategy should be to keep doses as low as reasonably achievable.

**Figure 2:**
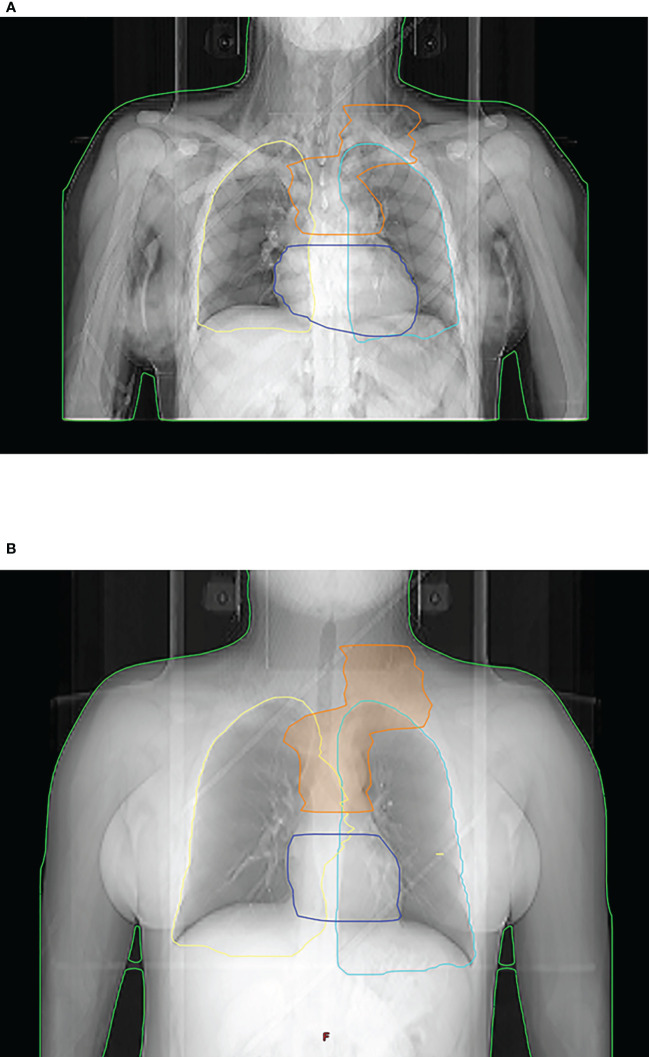
Radiation planning and treatment in free-breathing **(A)** vs. deep inspiration breath hold **(B)** in a young female patient. Inflation of the lungs due to inhalation reduces mean dose exposure both to the lungs and the heart.

To comply with the restrictive dose constraints, modern technologies like IMRT or RT in deep inspiration breath hold may be utilized (18). The latter describes a RT planning and treatment execution with inflated lungs mitigating dose exposure to the lungs and the heart (s. **Figure 2**). This technique has not been standard of care at the time of the HD17 trial, but should now be recommended for mediastinal PTV (9, 10). The technical evolution outlined is likely to impact dosimetric results with a further reduction of mediastinal OAR exposure in the modern era. Analysis of a larger patient collective in HD17 could elaborate a reduction in V20-V30 to the lungs with a concomitant increase in V5-V10 with modern IMRT (16). With an increasing percentage of IMRT today, low-dose exposure will rise, the impact of which is to be investigated in future analyses. Importantly, low-dose parameters like V5 are already considered in modern treatment guidelines and dose constraints (s. ILROG recommendations above).

Surprisingly, the use of INRT did not lead to a uniform reduction in mediastinal OAR exposure, a finding which may be explained by the rather large margins utilized for target volume definition (2-3 cm) (17).

Dosimetric analysis is not only a key factor to avoid overexposure to vulnerable organs at risk but also demands for a strict quality control of the treated target volumes. Inaccurate target volume coverage in HL has been identified as a risk factor for infield or marginal recurrence (26). In a historic analysis by Kinzie et al., incorrect field margins resulted in a recurrence rate of 50% in comparison to 15% in case of a correct setup (26). In accordance, the GHSG has a long-standing tradition of quality assessment and analysis for its treatment protocols (16, 27–34). Quality analysis of the HD4 demonstrated that relevant protocol violations resulted in a decline of relapse-free survival (7-year relapse-free survival: 72% with relevant protocol violations vs. 84% without; p=0.0043) (31). Future long-term analyses will show whether a similar relationship between quality of RT and oncological outcomes exists in the context of INRT and ISRT. Focusing on patients with early-stage unfavorable disease, a considerable improvement in quality could be outlined for the different study generations (RT series performed according to protocol: 33.0% in HD11 vs. 37.8% in HD14 vs. 74.4% in HD17) (16).

In the modern era, PET may help to establish new prognostic parameters like total lesion glycolysis (TLG) and total metabolic tumor volume (TMTV). The later has been shown to be a significant predictor both for PFS and overall survival in a *post-hoc* analysis of the H10 trial (35). In fact, TMTV was the only prognostic baseline parameter in contrast to conventional risk factors and enabled a downstaging of risk-category for > 70% of patients from intermediate/early-stage unfavorable to early-stage favorable disease (35). A comparable transition may be expected when applying this factor in the HD17 collective. In case of the HD16 trial, both TMTV and TLG were identified as predictors for the PET-result after two cycles of ABVD (36) which in turn determines the progression-free outcome (2).

The present analysis bears some limitations: despite all efforts, it was not possible to retrieve all DVH from the treating RT facilities. Not all DVH were transferred digitally or in a printed version eligible for dose extraction. Consequently, the RT plans used for evaluation represent only a fraction of all plans and it is unclear in how far this mirrors the overall picture within the study (and beyond). Furthermore, as a random sample was chosen for each treatment cohort, there was no case-control matching adjusting for further risk factors. This is especially true for the IFRT cohort, which has been limited in numbers intentionally. However, our previous dosimetric analysis showed no significant differences for most OAR limiting the bias (16). Another limitation is the widespread use of IFRT and older treatment techniques which could impair the transfer to modern radiotherapy practice. The HD17 trial was conducted prior to the widespread use of many advanced technologies like deep inspiration breath hold, limiting the generalizability of dosimetric data today.

The recent GHSG NIVAHL trial assessed the value of nivolumab in the first-line treatment of HL *via* a comparison between a sequential and a simultaneous chemo-immunotherapy approach (37, 38). Both arms revealed favorable outcomes with a PFS of 98% and 100%, respectively in the sequential and simultaneous cohort, respectively after a median follow-up of 41 months (37). With an obligatory 30 Gy involved-site RT as consolidation, this work also underlines the synergistic effect of RT in a modern multimodal treatment setting. As a result, rather than omitting RT in the therapeutic armamentarium, the goal of future trials should be to identify superadditive (systemic) combination partners and further personalize treatment fields. Emphasis has to be put on patients with extranodal disease or bulky disease as shown by our analysis. In the future, an individualized risk-benefit assessment will enable an evolution of RT-treatment strategies. Within the GHSG, this is currently pursued with the planned use of artificial intelligence incorporating large databases.

## Data availability statement

The data that support the findings of this study are available from the corresponding author, HTE, upon reasonable request.

## Ethics statement

The HD17 study protocol was reviewed and approved by the Ethikkommission - Medizinische Fakultät- Universität zu Köln. Subsequent approval was provided by the institutional review boards of the participating centers. Quality analysis of radiotherapy was an integral aspect of the study protocol. The patients/participants provided their written informed consent to participate in this study.

## Author contributions

The study was conceptualized by HE, with MO and HE being the principal investigators. MF and PB provided patient data from the study central database for analyses. Project management and statistical analysis was done by MO. CB, DV, RE-C, KH, PL, UH, MO and HE constituted the reference radiation panel and evaluated radiation plans. CK and DH extracted dosimetric data from dose-volume histograms and performed respective analyses. MO was responsible for data management. MO and HE wrote the first draft of the manuscript. All authors contributed to the article and approved the submitted version.
